# Neuronal and microglial mechanisms of neuropathic pain

**DOI:** 10.1186/1756-6606-4-31

**Published:** 2011-07-30

**Authors:** Min Zhuo, Gongxiong Wu, Long-Jun Wu

**Affiliations:** 1 Center for Neuron and Disease, Frontier Institute of Science and Technology, Xi'an Jiaotong University, Xi'an, China; 2 Department of Physiology, Faculty of Medicine, University of Toronto Centre for the Study of Pain, University of Toronto, 1 King's College Circle, Toronto, Ontario M5S 1A8, Canada; 3 Research Division, Joslin Diabetes Center, Harvard Medical School, Boston, MA 02215, USA; 4 Department of Neurobiology, Harvard Medical School and Department of Cardiology, Children's Hospital Boston, MA 02115, USA

## Abstract

Neuropathic pain is generally defined as a chronic pain state resulting from peripheral and/or central nerve injury. Effective treatment for neuropathic pain is still lacking, due in part to poor understanding of pathological mechanisms at the molecular level. Neuronal mechanisms of neuropathic pain, especially synaptic plasticity, are the major focus of many investigators. N-methyl-D-aspartate (NMDA) receptor dependent synaptic plasticity at the spinal and cortical levels is believed to contribute to enhanced sensory responses after injury. Glial cells, including astrocytes and microglia, have recently been implicated in neuropathic pain. These glial cells form close interactions with neurons and thus may modulate nociceptive transmission under pathological conditions. In this review, we present recent progress in the study of neuronal and microglial mechanisms underlying neuropathic pain. We propose that activity-dependent neuronal plasticity is a key target for treatment in neuropathic pain.

## Introduction

Pain is an unpleasant sensory experience induced by noxious stimuli. Physiological pain is important for animals to avoid potential injury, while pathological pain is unpleasant, lasts for an extended period of time after injury and is characterized by a heightened responsiveness to both noxious and non-noxious stimuli (hyperalgesia and allodynia, respectively). Neuropathic pain is generally defined as a chronic pain state resulting from peripheral or central nerve injury either due to acute events (e.g. amputation, spinal cord injury) or systemic disease (e.g. diabetes, viral infection and cancer). Chronic pain costs approximate $100 billion annually in healthcare and lost productivity in the United States [1]. Currently available treatments for neuropathic pain, including tricyclic antidepressants and the current "gold standard" gabapentin, typically show limited efficacy in the majority of patients [2].

To develop a better treatment for neuropathic pain, a comprehensive understanding of its pathogenesis is required. Chronic pain (such as inflammatory and neuropathic pain) is believed to be caused by aberrant neuronal responses along the pain transmission pathway from dorsal root ganglion (DRG) to, spinal cord, thalamus and cortex. Both peripheral and central origins are likely to be involved in chronic pain, although their contribution may be different depending on the various forms of chronic pain. For example, the sensitization of nociceptors after tissue injury by "inflammatory soup" leads to primary hyperalgesia and inflammatory pain. Similarly, central sensitization and synaptic plasticity in the central nervous system (CNS) contribute significantly to neuropathic pain. Therefore, targeting neuronal plasticity changes in somatosensory pathways is a major direction for finding pain relieving medications. However, it has been recently reported that neurons are not the only cell type involved in chronic pain states. Glial cells, including astrocytes and microglia, are emerging as possible additional players in the initiation and maintenance of neuropathic and inflammatory pain. These glial cells have close interactions with neurons and thus modulate pain transmission particularly under pathological conditions [3-5].

The aim of this review is to compare recent progress in neuronal and glial mechanisms underlying neuropathic and inflammatory pain. We focus on two major pain-related areas in the CNS, the spinal cord dorsal horn and anterior cingulate cortex (ACC). We will first examine the neuronal basis of chronic pain, and then review the recent progress in the role of glia in neuropathic and inflammatory pain, with particular emphasis on microglia. Finally, the cross-talk between neuronal and microglial mechanisms in neuropathic and inflammatory pain will be discussed.

### Neuronal mechanisms for neuropathic pain

Nociceptive signaling initiated in peripheral sensory neurons enters the spinal cord dorsal horn and is conveyed to supraspinal structures such as the brain stem, thalamus, somatosensory cortex, insular cortex and ACC [6]. Synapses within each relay are under precise regulation in order to provide appropriate behavioral responses. Integrative approaches including the use of human brain imaging and genetically manipulated mice have provided strong evidence for the suggestion that neuropathic pain is largely due to long-term plastic changes along sensory pathways. In the brain, activity-dependent synaptic plasticity is thought to be important for memory formation and storage [7]. In the nociceptive transmission pathway, plasticity underlies the cellular mechanism for behavioral sensitization in neuropathic pain. Plastic changes not only take place in peripheral nociceptors, spinal dorsal synapses, and subcortical nuclei, but also in cortical nuclei that are involved in the processing of noxious information. It is believed that neuropathic pain is likely due to long-term plastic changes along the nociceptive pathway [8]. It is likely that synaptic potentiation in the spinal cord and cortical areas together with abnormal peripheral activity after the injury contribute to neuropathic pain. Furthermore, basic mechanisms for sensory synaptic potentiation are often brain region dependent. We will discuss long-term plasticity in the spinal cord dorsal horn and the ACC to explore the neuronal mechanisms of neuropathic pain.

#### Synaptic plasticity in the spinal cord dorsal horn

The spinal cord dorsal horn is the first relay for pain transmission in the CNS. Glutamate is the principle fast excitatory transmitter and the corresponding postsynaptic responses are mediated by α-amino-3-hydroxy-5-methyl-4-isoxazole propionate (AMPA) and kainate receptors with a smaller contribution of N-methyl-D-aspartate (NMDA) receptors [9,10]. NMDA receptors serve as a key coincidence detector and are important for synaptic plasticity in central synapses. Therefore, it is believed that NMDA receptors play a critical role in injury-related synaptic plasticity in dorsal horn neurons, such as long-term potentiation (LTP) [11].

Sustained noxious stimuli that are associated with tissue injury in neuropathic pain result in a temporal summation of postsynaptic depolarization, which could relieve Mg^2+ ^blockade of the NMDA receptor. The activation of NMDA receptors would allow Ca^2+ ^influx, which in turn activates calcium-sensitive intracellular signal cascades that lead to the phosphorylation of the NMDA receptor and other receptor-ion channels, initiating prolonged increases in the excitability of spinal cord neurons [12]. Studies of LTP in spinal dorsal horn neurons have drawn much attention because it is believed that the potentiation of sensory responses after injury may explain neuropathic pain [13]. In *in vitro *spinal slices, LTP in the spinal dorsal horn neurons could be induced by several different protocols, including high frequency stimulation, low frequency stimulation or a pairing protocol. The mechanism of LTP induction involves the activation of NMDA receptors, neurokinin 1 receptors and the downstream MAP kinase pathway [14-16]. In addition, in vivo LTP of C-fiber-evoked responses could also be induced by low or high frequency stimulation of sensory nerve fibers [15]. Recent studies showed that NR2B-containing NMDA receptors are required for spinal LTP induction [17,18]. More importantly, in animals with the spinal cord and descending pathways intact, intraplantar injections of formalin or sciatic nerve jury induced LTP in the dorsal horn that was dependent on NMDA receptor activation [19,20].

Due to the unique gating properties of Mg^2+^, most NMDA receptors are not active under normal conditions. Thus, synapses containing only NMDA receptors are called silent synapses. Our results indicate the existence of silent synapses between sensory fibers in dorsal horn neurons. Moreover, 5-hydroxytryptamine (5-HT), an important neurotransmitter of the raphe-spinal projection pathway, transforms silent glutamatergic synapses into functional ones [21]. The mechanism underlying this conversion involves 5-HT induced PKC activation, AMPA receptor-PDZ interactions, and the recruitment of AMPA receptors [22]. Silent synapses are likely involved in synaptic potentiation in the spinal dorsal horn, considering that the recruitment of silent synapses could significantly enhance spinal sensory transmission, including nociceptive transmission. Another potential function of silent synapses is to contribute to a descending facilitatory modulatory network within the spinal cord. The recruitment by 5-HT could strengthen spinal sensory synapses receiving innervation from descending 5-HT projection fibers, which most likely originate from the rostral ventromedial medulla (RVM) [23].

In addition to synaptic potentiation of excitatory transmission in the spinal cord dorsal horn, recent studies showed that long-term disinhibition might also contribute to persistent pain. Peripheral nerve injury or inflammation leads to a long-lasting reduction in the expression of the potassium-chloride exporter KCC2, which increased the intracellular Cl^- ^concentration and thus alleviated the inhibitory effects of GABAergic or glycinergic transmission [24,25]. It is likely that such changes occur in a late phase of neuropathic pain and may play an important role in enhanced spinal excitability after injury. Upregulation of acid-sensing ion channels in the spinal cord dorsal horn has also been reported following peripheral inflammation [26-28]. It has been proposed that this upregulation of acid-sensing ion channels may contribute to spinal sensitization, and thus inflammatory pain [29].

#### Synaptic plasticity in the anterior cingulate cortex

Recent results from both human and animal studies are consistent with the suggestion that the ACC and related areas are important for pain perception. ACC neurons respond to nociceptive stimuli, and activity within the ACC is related to the unpleasantness or discomfort of somatosensory stimuli [30]. Electric stimulation or chemical activation of the ACC induced behavioral sensitization to heat or shock in animals [31,32], whereas blocking excitatory transmission or downstream cAMP and AC1&8 signaling inhibited pain-like behavior [33]. In addition, peripheral injury caused bilateral activity in the ACC, increased expression of immediate early genes, such as c-fos, Egr1 and CREB, and increased electrophysiological responses [34-36].

Electrophysiological experiments in cortical slices have shown that excitatory synaptic transmission in the cingulate cortex is primarily glutamatergic [37,38]. Both LTP and LTD of excitatory transmission can be induced in ACC slices [39-41], which allow a more detailed examination of the mechanisms underlying cortical plasticity. Both NR2A and NR2B NMDA receptors are required for LTP induction in the ACC. Multiple downstream signaling pathways such as calmodulin (CaM), calcium-stimulated AC1 and AC8, CaMKIV, and MAP kinase are involved in LTP in the ACC [40,42]. A series of studies from our laboratory has demonstrated that central plasticity occurs in the ACC, and is associated with chronic pain. Genetic forebrain overexpression of NR2B increased the NMDA-mediated postsynaptic current in the ACC and enhanced behavioral sensitization after inflammation [34]. We also found that peripheral inflammation caused the upregulation of NR2B in the ACC, which may underlie inflammation-related persistent pain [37]. Our recent results further showed that presynaptic release of glutamate and postsynaptic AMPA responses in ACC neurons are increased after peripheral nerve injury [43]. PKMzeta was found to be critical for maintaining synaptic potentiation in the ACC [44].

LTD might help to maintain appropriate neuronal activity within the ACC by reducing synaptic transmission. Using an amputation model and field recordings, we found that amputation of the third hindpaw digit in an adult rat caused a loss of LTD that persisted for at least 2 weeks [36]. The results of these studies are compatible with the hypothesis that synaptic LTD in the ACC enhances neuronal responses to subsequent somatosensory stimuli after amputation [35].

**Figure 1:**
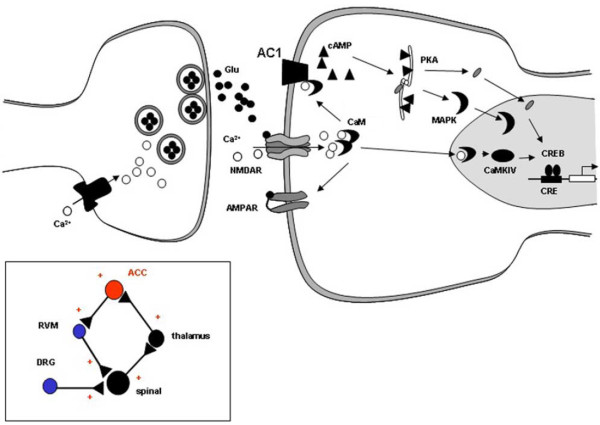
**Signaling pathways for NMDA receptor-dependent LTP in the ACC**. Neural activity releases glutamate in the ACC synapses. Subsequent to activation of glutamate NMDA receptors, Ca^2+ ^binds to CaM and leads to activation of calcium-stimulated ACs, including AC1 and AC8 and Ca^2+^/CaM dependent protein kinases (PKC, CaMKII and CaMKIV). Activation of CaMKIV, a kinase predominantly expressed in the nuclei, will trigger CaMKIV-dependent CREB. In addition, activation of AC1 and AC8 lead to activation of PKA, and subsequently CREB. CREB and other immediate early genes (e.g., Egr1) in turn activate targets that are thought to lead to more permanent structural changes. Inset box: the simplified neuronal network for sensory synaptic transmission, plasticity and regulation in the central nervous system. DRG: dorsal root ganglion.

A proposed synaptic model for the molecular mechanism of LTP in the ACC based on these studies is shown in Figure 1[11,45]. Neural activity triggered by injury increases the release of glutamate at the ACC synapse. The activation of NMDA receptors by glutamate leads to an increase in postsynaptic calcium in dendritic spines. Calcium binds to CaM, leading to the activation of calcium-stimulated signaling pathways. CaM stimulated ACs, including AC1 and AC8, CaM-dependent protein kinases, PKC, and CaMKII can all phosphorylate glutamate AMPA receptors, boosting their sensitivity to glutamate or increasing the number of synaptic receptors. Activation of CaMKIV, which is predominantly expressed in the nucleus, triggers CaMKIV-dependent CREB. In addition, activation of AC1 and AC8 leads to the activation of PKA and subsequently CREB. CREB, as well as other immediate early genes, activate targets that are thought to cause long-lasting changes in synaptic structure and function. Future studies are needed to map signaling pathways that contribute to the maintenance of LTP, including late-phase LTP, in the ACC.

#### Targeting NMDA receptors and synaptic plasticity for treatment of neuropathic pain

Current therapeutic strategies for neuropathic pain aim to reduce the excitability of neurons by modulating ion channel activity (such as gabapentin and lidocaine) or by enhancing endogenous inhibitory mechanisms (such as tricyclic antidepressants and opioids). As we discussed above, NMDA receptors play a critical role in synaptic plasticity within pain transmission pathways and are thus likely to be important in neuropathic pain. However, among clinically assessed NMDA antagonists, the narrow separation between effectiveness and liabilities, such as sedation, memory impairments, motor incoordination and psychotomimetic effects, has severely hampered their utility for the treatment of neuropathic pain. NR2B subunit-containing NMDA receptors are localized in pain-relevant structures, such as in superficial layers of the dorsal spinal horn, thalamus, hippocampus and cortex. The restricted distribution of NR2B makes it promising as a candidate target of side effect-free analgesic drugs. Cited By in Scopus (7)Indeed, NR2B antagonists, such as ifenprodil and related compounds, are effective in neuropathic pain in animals, and show better separation between efficacy and side effects in human patients than non-selective NMDA receptor blockers [2,46]. Importantly, these drugs are non-addictive and may even attenuate morphine-induced conditioned place preference [47]. Therefore, NR2B NMDA receptors and their downstream signaling targets such as AC1 are potential targets for neuropathic pain [11,46,48].

### Glial mechanisms for neuropathic pain

Our understanding of pathological pain has evolved from solely neuronal mechanisms to neuron-glial interactions. In particular, astrocytes and microglia act as possible modulators of neuropathic pain by releasing a number of cytokines and chemokines. Astrocytes and microglia play different roles in relation to neuronal activity; however, they do have some overlapping functions in mediating CNS innate immune response [5]. Both astrocytes and microglia are activated in neuropathic pain, and their activation leads to pro-inflammatory responses with pathological effects, such as neuronal hyperexcitability, neurotoxcity and chronic inflammation. The roles of astrocytes in neuropathic pain have been summarized in a number of recent reviews [5,49]. Here we will focus on the role of microglia in neuropathic pain

#### Mapping microglial cells in the CNS

Microglia are the resident macrophages and principal immune-response cells in the CNS [50]. They comprise 5-10% of the glial cell population and are quite evenly distributed in the brain. Little is known about the function of resting microglia under normal conditions in the brain. Recently, it was found that resting microglia have highly dynamic processes and survey the microenvironment in the brain in vivo [51,52] or in acute brain slices in vitro [53]. Under pathological conditions, these cells are activated and exhibit chemotactic, phagocytoxic and secretory responses to various stimuli [54]. Immunostaining for microglia-specific antigens, such as Iba1, OX-42, CD11b, CD4, ED1 or major histocompatibility complex (MHC) II are commonly used for microglial identification in situ [55]. To study microglial functions like migration, phagocytosis, or cytokine release, cultured microglia are often used. A Boyden chamber or Dunn chamber is commonly used for migration assays [56], while beads, bacteria, or neurons labeled with fluorescent markers are used for phagocytosis. Microglia in culture may be transformed into amoeboid cells, which do not represent resting microglia in situ. Recently, in vivo two photon imaging techniques were applied to monitor brain microglia activities [51,57]. Although technical limitations hampered further study of the molecular mechanisms underlying microglial responses, especially for those located in deeper brain areas, in vivo imaging provides a unique chance to examine brain microglial motility and neuron-microglia communication under physiological and pathological conditions.

**Figure 2:**
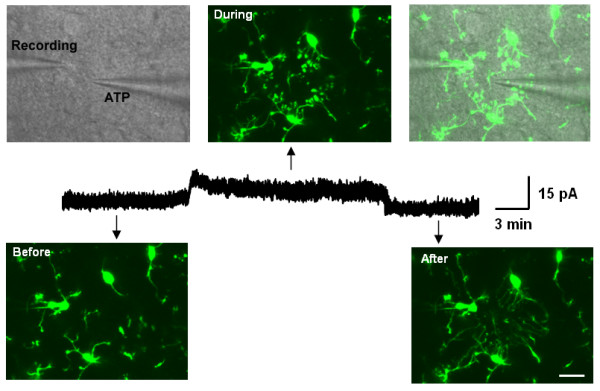
**Simultaneous confocal imaging and patch clamp recordings in microglia in situ**. ATP-containing pipette induced both outward current and chemotaxis in brain microglia. Outward current appears after pipette approaches, while disappears after the pipette moves away from the recording microglia (middle). DIC image showed the recording pipette and ATP-containing pipette. Fluorescent images showed that the chemotactic effect during the insertion of the ATP pipette (upper). When the pipette was removed out of the slice, the moving terminals rapidly disappeared (lower, right). Scale bar, 15 μm. Modified from [53].

Electrophysiology has been used to study microglial membrane receptors and ion channels in recent years. Pioneering work on microglia electrophysiology was done by the Kettenmann group, showing that microglia express currents mediated by a variety of ion channels and neurotransmitter receptors in vitro and in situ [58]. Most studies on microglia electrophysiology have been conducted in cultured cells. Only a few studies have been performed in young brain slices, in part due to the difficulty of identifying microglial cells in brain slices [59]. The recent development of transgenic mice with green fluorescent protein (GFP)-labeled microglia provides a good tool to study microglia in situ [60,61]. Taking advantage of these transgenic mice, one could easily identify microglia and perform patch clamp recordings in acute brain slices. These microglia largely represent the properties of resting microglia in both morphology and electrophysiology [53,62]. Simultaneous time-lapse confocal imaging and perforated whole-cell recordings were also applied to study microglial electric responses during chemotaxis [53]. For example, we have shown that ATP can induce a rapid chemotaxis of microglial processes (in minutes), which is associated with the generation of an outward K^+ ^current (Figure 2). The results provide a novel insight into ATP receptors and their associated signaling pathways in the control of microglial movement. This imaging method was also applied in the spinal cord dorsal horn and a similar ATP-induced chemotaxis was observed [63].

#### Activation of spinal but not cortical microglia in neuropathic pain

Resting ramified microglia rapidly transform into an activated state in most pathological conditions, including host defense against infectious organisms, autoimmune inflammation, ischemia, trauma, neurodegeneration and neuropathic pain [50,64]. Activation of microglia is accompanied by changes in morphology, upregulation of immune surface antigens and the production of cytotoxic or neurotrophic molecules [54,65]. Under neuropathic pain conditions, microglial activation in the spinal dorsal horn has been demonstrated by at least three different methods. First, the morphology of microglia transforms from "resting" states, which have thin and branched processes, to "activated" states, which are characterized by hypertrophy with thickened and retracted processes [64]. The morphological change is also associated with proliferation or microgliosis. For example, it has been shown that dorsal horn laminae I to III manifested a higher density of microglia after peroneal nerve ligation [66]. Second, compared with resting microglia, these activated microglia exhibit a change in surface markers, membrane-bound or embedded proteins. These include CD11b, P2X4 receptors, toll-like receptor 4, CD44, and MHC II [3]. Third, the activation of spinal microglia in neuropathic pain is characterized by phosphorylation of MAP kinases, including the p38 and Src-family kinases, such as Lyn [3,67]. Electrophysiology has not been applied to study activated microglia in the spinal dorsal horn associated with neuropathic pain; it has been reported that in the facial nucleus, after nerve axotomy, microglia exhibit prominent inward rectifier currents [68]. Interestingly, spinal microglia activation occurs during the early phase of neuropathic pain and precedes astrogliosis, supporting the current hypothesis that microglia may be important for initiation, while astrocytes are important for the maintenance of neuropathic pain [67,69]. It is important to point out that none of the spinal glial cells project to the brain. Thus, the influence of glial changes may act through ascending neuronal transmission. Unfortunately, no recent studies address this critical aspect of neuropathic pain.

**Figure 3:**
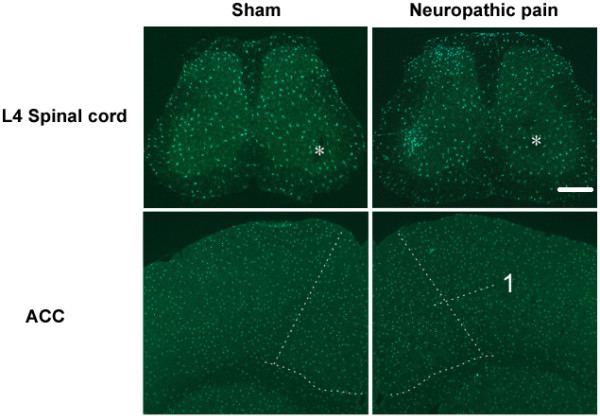
**Spinal but not cortical microgliosis after peripheral nerve injury**. Left column, sham-operated; right column, peripheral nerve injury. Nerve injury evoked an ipsilateral increase of microglial cells in L4 spinal dorsal horns. Asterisks indicate contralateral side of nerve injury. The structures in the cortex are indicated by arrow or enclosed by dashed lines. 1, ACC (Anterior cingulate cortex). Bar = 350 μm. Modified from [66].

Using transgenic mice in which microglia are selectively labeled with GFP, we have recently performed systematic mapping of microglia in major pain-related brain areas in mice following nerve injury [66]. Although we have confirmed the activation of spinal microglial cells after nerve injury, we did not find any microgliosis in supraspinal structures, including the somatosensory cortex and the ACC (Figure 3). These findings are consistent with our recent study demonstrating that microglial motility was not altered by neuronal activity or LTP induction in the aforementioned brain regions [62]. In spite of this lack of microgliosis, we cannot completely exclude the possibility that microglia are altered at the biochemical and molecular levels in neuropathic pain. Indeed, upregulation of microglial and astrocytic markers such as OX-42 and GFAP was observed in rat brain after peripheral administration of complete Freunds adjuvant (CFA) to produce inflammation [70]. Interestingly, astrogliosis was observed in the ACC after sciatic nerve ligation [71].

#### Mechanism for the activation of microglia in neuropathic pain

Spinal microglia are activated in the setting of neuropathic pain. However, signals that mediate microglial activation are still poorly understood. In the setting of neuropathic pain, peripheral neurons transmit signals to spinal dorsal horn neurons, releasing neurotransmitters such as calcitonin gene-related protein (CGRP), substance P, glutamate, and ATP. Locally in the dorsal horn, there are also other neurotransmitters involved, such as GABA, glycine, serotonin. Therefore, it is plausible to suggest that these neurotransmitters may initiate microglial activation associated with neuropathic pain. However, by using whole-cell patch clamp recordings, we found that resting microglia did not show any observable current using electric stimulation, exogenous glutamate or GABA [62]. Consistently, local applications of neurotransmitters such as glutamate, GABA, and substance P, neuromodulators and chemokines such as serotonin, noradrenaline, carbachol, CX3CL1 (fractalkine), MCP-1, and interleukins (IL), or electric stimulation of dorsal root fibers with noxious intensity did not induce microglial chemotaxis in the spinal cord dorsal horn [63]. Therefore, it seems that resting microglia do not have fast electrical or chemotactic responses to these neurotransmitters or cytokines. Nevertheless, microglia may still sense these neuronal signals via other means.

Microglia respond to several neuronal-derived signals which may lead to microglial activation after peripheral nerve injury. These signaling pathways include ATP and its receptors (P2X and P2Y receptor), fractalkine and CX_3_CR1, monocyte chemotactic protein (MCP-1) and CCR2. Spinal microglia have robust responses to ATP via purinergic signaling. Similar to microglia in the brain, spinal microglia show fast chemotaxis in response to local application of ATP [63]. Microglia are known to express both ionotropic receptors, such as P2X4 and P2X7, and metabotropic receptors, such as P2Y6 and P2Y12 [72,73]. Activation of P2X4 in microglia facilitates BDNF release [74], while activation of P2X7 in microglia induces IL1β release and CXCL2 production [75,76]. Interestingly, The P2Y12 receptor in microglia is reported to mediate ATP-induced microglial chemotaxis [53,77] while P2Y6 may mediate microglial phagocytosis [78]. Moreover, ATP induced both inward and outward current in resting microglia, which may be mediated by P2X and P2Y receptors, respectively [53]. In models of neuropathic pain, both P2X4 and P2Y12 receptors are upregulated in microglia, but not in neurons or astrocytes in the dorsal horn. Pharmacological inhibition or genetic deletion of either P2X4 or P2Y12 alleviates allodynia or heat hyperalgesia after peripheral nerve injury [79,80].

Fractalkine and its receptor CX_3_CR1 are also involved in microglial activation associated with neuropathic pain. Fractalkine is a neuronal transmembrane glycoprotein that can be released after being cleaved by proteolysis. Although fractalkine did not induce microglial chemotaxis in vitro, intrathecal injection of fractalkine activates p38 in spinal microglia and produces mechanical allodynia and thermal hyperalgesia [81]. The cleavage of fractalkine may involve cathepsin S, a cysteine protease that is expressed in spinal microglia. It has been shown that noxious stimulation of primary afferent fibers induces release of cathepsin S from microglia; the process may require P2X7 activation [82,83]. Intrathecal application of cathepsin S also induces p38 activation and allodynia [84]. Microglial cells constitutively express CX_3_CR1, and its expression is markedly upregulated in models of neuropathic pain [81,85]. Therefore, it is conceivable that fractalkine may act on CX_3_CR1 to exert its effects in neuropathic pain. However, most studies only used indirect behavioral studies to test this possibility. For example, microglial p38 activation and behavioral sensitization produced by fractalkine or cathepsin S are largely reduced in either CX_3_CR1 knockout mice or after using a neutralizing antibody against CX_3_CR1 [81,84,86]. A recent study also confirmed that there is reduced inflammatory and neuropathic pain, and a decreased spinal microglial response in CX_3_CR1 knockout mice [87].

In addition, MCP-1 and its receptor CCR2 are involved in microglial activation and neuropathic pain. Intrathecal injection of MCP-1 produced tactile allodynia, while a MCP-1 neutralizing antibody reduced neuropathic pain [88]. The expression of MCP-1 is induced in primary sensory neurons after peripheral nerve injury and it may be transported to the central terminals of primary afferents in the dorsal horn [88,89]. Microglial cells in the dorsal horn express CCR2, which may mediate MCP-1's effect, as well as neuron-microglia communication in neuropathic pain. Consistently, MCP-1 induced microglial morphology transitions and p38 activation in microglia. Additionally, neuropathic pain is substantially reduced in mice lacking CCR2 [90,91].

In addition to these three major pathways that mediate microglial activation in models of neuropathic pain, other molecules may also be important, including toll-like receptors, MHC class II protein, and CB2 receptors [3]; however, their endogenous ligands in the setting of neuropathic pain remain to be clarified. Recent progress also points to the potential involvement of microglial INFγ receptors, ErbB receptors, and NADPH oxidase 2 in microglial activation and neuropathic pain [92-94].

#### Activated microglia and neuropathic pain

Whether or not activated microglia are sufficient to cause neuropathic pain is still open to debate. Intrathecal administration of microglia, activated by ATP, decreased the pain threshold, while a similar application of activated astrocytes did not [80,95]. This result supports the suggestion that activated microglia are sufficient to produce neuropathic pain. However, other studies have found a dissociation of microglial activation and tactile allodynia [96,97].

How might activated microglia contribute to neuropathic pain? One intriguing pathway involving ATP and the P2X4 receptor has been proposed. Peripheral nerve injury leads to an upregulation of P2X4 receptors in activated microglia. ATP acts on the P2X4 receptor in microglia and induces intracellular Ca^2+ ^elevation and phosphorylation of p38, which subsequently increase BDNF synthesis and release [74]. BDNF released from microglia may produce a depolarizing shift in the Cl^- ^reversal potential by reduction in the expression of the potassium-chloride exporter KCC2 in dorsal horn neurons [98]. The shift in anion reversal potential prompts a disinhibition and thus facilitates mechanical allodynia after nerve injury.

In animal models of neuropathic pain, activated microglia also increase synthesis and secretion of various cytokines and chemokines, including IL-1β, IL-6, tumor necrosis factor-α(TNFα), PGE2, and nitric oxide. It has been shown that p38 activation turns on the transcription factor NF-κB, which leads to the expression of IL-1β, IL6, and COX-2 [99]. These cytokines, released by activated microglia, will amplify microglial activation in an autocrine manner and may act directly on dorsal horn neurons to cause behavioral sensitization. For example, it has been shown that intrathecal administration of IL-1β, IL6, or TNFα can lead to symptoms of neuropathic pain in healthy rats [100]. However, direct evidence for enhanced spinal sensory transmission is still lacking, despite numerous pharmacological and behavioral studies [69]. Many key discoveries still need to be confirmed at the cellular and synaptic levels. Relying solely on behavioral results can be misleading, as they are easily affected by the environment, modulatory effects on inhibitory neurons, and side effects on motor function.

#### Targeting microglia for neuropathic pain

Given the increasing evidence that microglia play a critical role in the development of neuropathic pain, microglia and related signaling molecules hold promise as targets for pain control. The therapeutic benefits of targeting microglial molecules may reside in the fewer side effects on acute pain sensation, as most of these molecules are upregulated predominantly in activated microglia. A few immunosuppressive compounds are being developed to attenuate microglial activation and inflammation and have proven to be effective in animal models of neuropathic pain [101]. For example, minocycline, a second-generation tetracycline antibiotic, selectively targets microglia to globally inhibit metabolism. At a cellular level, this drug suppresses the expression of inducible nitric oxide synthase, the production of pro-inflammatory cytokines, and p38 phosphorylation in microglia; at the behavioral level, it is therapeutically effective in animal models of neuropathic pain [102]. Minocycline can cross the blood-brain barrier and is under clinical investigation as a treatment for multiple sclerosis and amyotrophic lateral sclerosis [103]. However, it should be noted that minocycline also has a variety of side effects, including fever, stomach pain, effects on vision, drowsiness, and even psychiatric problems [104]. Therefore, future studies are needed to elucidate the molecular mechanisms of minocycline's effects on brain microglia as well as other cells. Other drugs targeting inflammation include ankinra (an antagonist of IL-1β) and etanercept (a TNF receptor fusion protein). Both demonstrated beneficial effects in animal models of neuropathic pain [105,106].

### Microglia and inflammatory pain

Recent studies have shown that activation of microglial cells in the spinal cord may contribute to inflammatory pain. For example, Clark et al (2007) reported ipsilateral dorsal horn microglial activation 2 weeks after injury, and bilateral activation 50 days following intraplantar zymosan, but not intraplantar CFA. They found that spinal injection of the glial metabolic inactivator fluorocitrate reversed hyperalgesia after intraplantar zymosan and produced no reversal of CFA-induced hyperalgesia [107]. Therefore, the involvement of microglia in inflammatory pain is dependent on the inflammatory stimulus administered.

The involvement of microglia in inflammatory pain may also happen at a supraspinal level. Microinjection of the glial inhibitors minocycline or fluorocitrate into the RVM produced a significant and time-related attenuation of behavioral hypersensitivity resulting from hindpaw inflammation [108]. Carrageenan-induced inflammation increased immunolabeling of microglia and astrocytes in the RVM, suggesting that inflammatory pain is associated with glial activation in the RVM [108]. Recently, it was reported that inflammatory and neuropathic nociceptive responses were reduced in CX3CR1 knockout mice, although the possible contribution of spinal *versus *brainstem glial cells has not been examined in the same study [87]. Most of these studies used pharmacological and behavioral approaches. Future studies using electrophysiological methods are needed to confirm the effects of inhibitors or gene deletion on pain transmission. It is important to exclude any possible modulatory effects on motor functions or sensory related motor circuits.

### Cross-talk between neuronal and glial mechanisms

As we have described in our discussion of the neuronal mechanisms of neuropathic pain, long-term plastic changes along sensory pathways play a central role in mediating behavioral sensitization. On the other hand, glial cells such as microglia are also critical for the development of neuropathic pain. These two parallel causes of neuropathic pain are not independent, acting in concert to generate a behavioral pain phenotype.

In the spinal dorsal horn, it has been reported that microglial activation may partially contribute to the induction of LTP. The mechanism may involve the activation of a microglial ATP receptor, such as P2X4 or P2X7, p38 phosphorylation and IL-1 release [109,110]. The exact synaptic mechanism for potentiation, however, remains to be determined. In contrast to the spinal cord, microglial activation in brain inflammation and infection are associated with attenuations in LTP in the hippocampus during aging [111], chronic brain inflammation [112] or human immunodeficiency virus infection [113]. The discrepancy may be due to a wide range of immune mediators released from activated microglia, some of which are destructive, while others exert protective effects on neurons and pain transmission.

Activity-dependent synaptic plasticity is associated with the release of various neuromodulators, such as ATP, BDNF and tissue plasminogen activator (tPA) [114-116], which may affect microglia. However, we have found that high or low frequency stimulation of dorsal roots, which could induce long-term plasticity in the spinal dorsal, did not affect microglial motilities [63]. Similarly, microglial motility is independent of LTP in brain slices [62]. Although these results did not exclude the possibility that LTP induction may cause biochemical changes in microglia, which mimic microglial activation during pathological insults, they provide new insights into the role of resting microglia in physiological functions compared to pathological conditions. Furthermore, in the ACC, despite enhanced excitatory transmission between neurons, no microgliosis was found after peripheral nerve injury [43,66]. It is thus likely that neuronal plasticity plays a key role in injury-related changes in the ACC.

### Conclusions and future directions

After two decades of searching, there is still a lack of effective treatments for neuropathic pain. Clearly, more basic research is needed at the molecular, cellular, systemic, and behavioral levels. Understanding neuronal and glial mechanisms in neuropathic pain offers hope of developing painkillers targeting NMDA receptors or microglia-related molecules. One obvious challenge for most treatments is their side effects on cognition. NMDA receptors play important roles in various cognitive functions, while microglia survey the microenvironment in the brain. Therefore, the use of such drugs for neuropathic pain is likely to require balancing a gain and loss of brain function. It may be inevitable that some cognitive functions must be sacrificed in order to control severe pain in some patient populations. Research is needed that focuses on the following routes to develop painkillers with an improved therapeutic index: (1) Targeting NR2B-selective NMDA receptors. NR2B antagonism has been shown to be effective in neuropathic pain treatment, with fewer side effects than traditional therapies. More efforts should be made to develop selective NR2B antagonists and test them in preclinical and clinical trials; (2) Targeting forebrain NMDA receptors. Recent studies from both human and animal consistently demonstrate that forebrain areas are important for processing pain perception and the unpleasantness of somatosensory stimuli. Therefore, selectively targeting NMDA receptors in the forebrain would provide new insights into treatment of neuropathic pain [11]; (3) Targeting proteins downstream of the NMDA receptor, such as calcium-stimulated AC1 and other protein kinases. We hope that the basic mechanisms of neuropathic pain elucidated from animal studies will help us to understand why some drugs are ineffective treatments for neuropathic pain; we also hope that the identification of new protein targets such as AC1 will facilitate the development of new medicines for chronic pain [48].

## Competing interests

The authors declare that they have no competing interests.

## Authors' contributions

MZ and LJW conceived of the study, MZ, GW and LJW drafted the manuscript. All authors read and approved the final manuscript.
